# Efficacy and safety of a fixed-dose oral tramadol–dipyrone combination for postoperative analgesia in dogs undergoing ovariohysterectomy and unilateral mastectomy: a randomized clinical trial

**DOI:** 10.3389/fvets.2026.1898549

**Published:** 2026-08-21

**Authors:** Sandra Mastrocinque, Karina Velloso Braga Yazbek, Richard Barrientos Rossetti, Laura Damião Gomes, Jeovan dos Santos Macedo, Amanda Cologneze Brito, Érica Vilela Barreto, Denise Tabacchi Fantoni

**Affiliations:** 1 Leais Vet, Ribeirão Preto, Brazil; 2 FAMESP–Faculdade Método de São Paulo, São Paulo, Brazil; 3 Technical Department, Clinical Research and Development of New Products, Biolab Sanus Farmacêutica Ltda, Taboão da Serra, SP, Brazil; 4 UTI Vet, Ribeirão Preto, Brazil; 5 Atuare Care – Veterinary Specialty Center, Ribeirão Preto, Brazil; 6 Department of Surgery, School of Veterinary Medicine and Animal Science, University of São Paulo, São Paulo, SP, Brazil

**Keywords:** dogs, mastectomy, metamizole, multimodal analgesia, ovariohysterectomy, postoperative analgesia, rescue analgesia, tramadol

## Abstract

**Introduction:**

To evaluate the analgesic efficacy and safety of an oral tramadol–dipyrone combination (Sindolor^®^) compared with tramadol or dipyrone alone in dogs undergoing ovariohysterectomy and unilateral mastectomy.

**Methods:**

Thirty client-owned female dogs undergoing elective ovariohysterectomy and unilateral mastectomy were randomly assigned to three treatment groups (n = 10/group): tramadol–dipyrone combination (GTD), tramadol alone (GT), or dipyrone alone (GD). At the end of surgery, all dogs received meloxicam and the first intravenous dose of the assigned treatment. Oral treatment began approximately 8 h later and continued every 8 h for 5 days. Postoperative pain was assessed using the Glasgow Composite Measure Pain Scale–Short Form (GCMPS-SF), a dynamic interactive visual analog scale (DIVAS), and serum cortisol concentrations. Rescue analgesia with intravenous morphine was administered when predefined thresholds were reached.

**Results:**

Pain scores decreased significantly over time in all groups (*p* < 0.01). Despite concurrent meloxicam administration, dogs receiving dipyrone alone showed higher GCMPS-SF scores than those treated with the tramadol–dipyrone combination at D + 1 afternoon (*p* < 0.01). Pain incidence analysis demonstrated a higher proportion of pain-free dogs in the combination group during the early postoperative period. Overall rescue analgesia frequency did not differ significantly among groups; however, after initiation of oral treatment, rescue analgesia was required in two dogs from both GT and GD, whereas no dog in GTD required additional analgesia. Cortisol concentrations remained within the reference range in GTD, whereas transient increases above the reference interval were observed in GT and GD. No clinically relevant adverse effects were observed.

**Conclusions and clinical relevance:**

The tramadol–dipyrone combination improved early postoperative pain control in dogs undergoing ovariohysterectomy and unilateral mastectomy while maintaining favorable tolerability. These findings support its clinical use as part of a multimodal postoperative analgesic protocol, particularly during the early postoperative period.

## 1 Introduction

Postoperative pain remains a major clinical concern in veterinary medicine, as inadequate analgesia may impair recovery, delay return to normal function, and negatively affect animal welfare (1). Effective pain management is therefore a fundamental component of perioperative care, particularly in procedures associated with moderate to severe tissue injury (2).

Multimodal analgesia is widely recommended for postoperative pain management because combining analgesics with different mechanisms of action may improve analgesic efficacy while reducing the required doses of individual drugs and minimizing adverse effects (3). In dogs, the selection and escalation of analgesic protocols are generally guided by the expected intensity of postoperative pain, with more invasive or highly painful procedures requiring broader multimodal strategies. Clinical evidence supports this approach; for example, the combination of opioids and non-steroidal anti-inflammatory drugs (NSAIDs) has been associated with improved postoperative analgesia and reduced rescue analgesic requirements in dogs undergoing ovariohysterectomy (4). Accordingly, multimodal protocols are considered particularly important when managing procedures associated with moderate to severe nociceptive pain.

In dogs, the analgesic efficacy of tramadol remains controversial. This controversy is largely attributed to the limited formation of its active metabolite O-desmethyltramadol (M1), resulting in reduced *μ*-opioid receptor activation compared with humans (5, 6). Nevertheless, tramadol also exerts analgesic effects through inhibition of serotonin and norepinephrine reuptake, and differences in pain models, multimodal analgesic protocols, study design, and outcome assessment methods may further contribute to the inconsistent findings reported in the veterinary literature. Despite these limitations, tramadol is commonly incorporated into multimodal analgesic protocols in veterinary medicine, particularly when oral analgesia is required (7, 8). Several clinical and experimental studies support its role as part of multimodal analgesic strategies. In dogs, tramadol has demonstrated postoperative analgesic effects in surgical settings, and its adjunctive use with other analgesics has also shown beneficial outcomes in chronic pain conditions such as osteoarthritis (8, 9). These findings support the rationale for evaluating tramadol as a component of combination analgesic protocols.

Metamizole (Dipyrone) is a non-opioid analgesic widely used in humans and in veterinary patients in several countries because of its analgesic, antipyretic, and antispasmodic properties, particularly in perioperative and acute pain settings (10, 11). Its mechanisms of action are not yet fully elucidated and likely involve both central and peripheral pathways. In clinical practice, dipyrone is frequently incorporated into multimodal analgesic protocols, particularly when additional non-opioid analgesia is desired.

Evidence supporting this combination has been described in human medicine and experimental models, suggesting additive or synergistic analgesic effects. In rodents, tramadol has been shown to enhance the antinociceptive effects of dipyrone, potentially through pharmacodynamic interactions that improve analgesic response (12). More recently, a randomized blinded clinical study in cats undergoing ovariohysterectomy demonstrated that the oral fixed combination of tramadol and dipyrone provided effective postoperative analgesia with an acceptable safety profile (13). However, clinical evidence evaluating this association in dogs remains limited.

Assessment of postoperative pain in dogs remains challenging because behavioral responses may be influenced by individual temperament, stress, environmental factors, and observer interpretation. For this reason, validated composite pain assessment tools are essential for clinical and research settings. In addition to behavioral assessment, physiological or endocrine markers such as serum cortisol may provide complementary information regarding perioperative stress responses, although they should not be interpreted as isolated indicators of pain (12).

Unilateral mastectomy combined with ovariohysterectomy is recognized as a painful surgical model associated with moderate to severe postoperative nociception, making it suitable for the evaluation of analgesic interventions. Therefore, the objective of this study was to evaluate the postoperative analgesic efficacy and safety of an oral fixed combination of tramadol and dipyrone, administered as part of a multimodal postoperative analgesic protocol, in dogs undergoing unilateral mastectomy and ovariohysterectomy, compared with tramadol or dipyrone administered alone over a 5-day postoperative period.

## 2 Materials and methods

### 2.1 Animals and study design

A randomized, blinded, comparative study design was used. Thirty client-owned female dogs of various breeds, aged 2 to 10 years, were included. Dogs were randomly assigned to one of three treatment groups using a computer-generated allocation sequence (Microsoft Excel) prepared by an investigator not involved in postoperative data collection or outcome assessment. Personnel responsible for treatment preparation and administration did not participate in postoperative evaluations. Clinical assessments and data collection were performed by a blinded team using coded treatment groups, and group allocation was revealed only after completion of the statistical analyses.

All dogs were diagnosed with mammary neoplasia based on physical examination. Although some dogs presented unilateral and others bilateral mammary chain involvement, all underwent standardized unilateral mastectomy to ensure a comparable nociceptive surgical stimulus across animals. Dogs requiring additional surgical intervention subsequently underwent further procedures as clinically indicated.

Owners provided written informed consent for participation of their animals in the study. In addition, owners were informed that animals would remain hospitalized for 5 days following surgery, and consent for this postoperative monitoring period was also obtained. This hospitalization period was considered necessary to ensure appropriate clinical recovery, standardized pain assessment, and close monitoring for potential adverse effects under controlled conditions. During this time, animals were housed in a clinical environment with continuous supervision. All procedures and postoperative care were performed at Veterinary Clinic Castelo, Ribeirão Preto, Brazil.

The study was conducted in accordance with the Brazilian guidelines for animal experimentation established by the National Council for the Control of Animal Experimentation (CONCEA) and was approved by the institutional animal care and use committee (Protocol number 1106/20). The study was reported in accordance with the CONSORT guidelines, adapted where applicable for clinical studies in client-owned animals.

Exclusion criteria included renal or hepatic dysfunction based on laboratory evaluation (urea, creatinine, alkaline phosphatase, and alanine aminotransferase concentrations), abnormalities in complete blood count (including red and white blood cell and platelet counts), and systolic arterial pressure below clinically acceptable limits measured using an oscillometric device (DX2710, Dixtal, São Paulo, Brazil). Animals with cardiovascular disease, arrhythmias, or poor general health status were also excluded. Dogs receiving opioid analgesics or anti-inflammatory drugs within 7 days prior to surgery were not eligible for inclusion. Animals showing marked signs of stress, fear, or aggressive behavior that could interfere with handling, welfare, or the reliability of pain assessment were also excluded, as well as pregnant or lactating females.

Baseline measurements of heart and respiratory rate, arterial blood pressure, and venous blood samples were obtained 30 min prior to premedication. Dogs were admitted 24 h before surgery for acclimatization.

### 2.2 Anesthesia and surgical procedures

Food and water were withheld for 8 and 2 h, respectively. Dogs were premedicated with acepromazine (0.01 mg/kg; Acepran^®^ 0.2%, Vetnil Indústria Comércio Produtos Veterinários Ltda, SP, Brazil), and meperidine (4 mg/kg; Dolosal^®^, Cristália Produtos Químicos e Farmacêuticos Ltda, SP, Brazil) intramuscularly. The cephalic vein was catheterized, and lactated Ringer’s solution was administered at 5 mL/kg/h during surgery. Anesthesia was induced 15 min later with propofol (4–5 mg/kg; Propovan^®^, Cristália Produtos Químicos e Farmacêuticos Ltda, SP, Brazil), administered intravenously and titrated to effect, in combination with ketamine (0.5 mg/kg; Cetamin^®^, Syntec do Brasil). After orotracheal intubation, anesthesia was maintained with isoflurane (1.2–1.4 V%; Isoflorane^®^, Cristália Produtos Químicos e Farmacêuticos Ltda, SP, Brazil) in 100% oxygen using a rebreathing system, with animals remaining in spontaneous ventilation unless end-tidal CO₂ exceeded 40 mmHg.

Following stabilization, all dogs received fentanyl (2.5 μg/kg IV bolus; Fentanest^®^, Cristália Produtos Químicos e Farmacêuticos Ltda, SP, Brazil) followed by continuous infusion (10 μg/kg/h). Hypotension was treated with fluid bolus (10 mL/kg over 10 min) and, if necessary, ephedrine (0.25 mg/kg IV; Efedrin^®^, Cristália Produtos Químicos e Farmacêuticos Ltda, SP, Brazil). A rescue bolus of fentanyl (1.0 μg/kg IV) was administered in response to increases of ≥15% in heart rate or arterial blood pressure relative to values obtained after anesthesia stabilization.

All surgical procedures were performed by the same senior surgeon to minimize procedural variability.

### 2.3 Analgesic protocol and postoperative evaluation

Pain were assessed by a blinded evaluator (S. M.), with extensive experience in veterinary pain assessment, who remained unaware of treatment allocation throughout the study.

Approximately 10 min before the end of surgery, dogs received the first dose of the assigned treatment according to group allocation: tramadol–dipyrone fixed combination (GTD; tramadol 2 mg/kg + dipyrone 25 mg/kg), tramadol alone (GT; 2 mg/kg), or dipyrone alone (GD; 25 mg/kg), intravenously. At extubation, all animals received meloxicam (0.2 mg/kg SC; Meloxinew^®^, Vetnil Indústria Comércio Produtos Veterinários Ltda, SP, Brazil) as part of the standard multimodal postoperative analgesic protocol. Oral administration of the assigned treatments began 8 h after the initial intravenous dose and continued every 8 h for 5 consecutive postoperative days, while meloxicam was maintained orally at 0.1 mg/kg once daily during the same postoperative period. Oral treatments were administered using commercially available formulations: tramadol (Cronidor^®^, Agener União, SP, Brasil), dipyrone (Dipirona^®^, Biovet, SP, Brasil), and the fixed-dose new combination tablets (Sindolor^®^, Avert Saúde Animal, SP, Brasil). Doses were adjusted to the nearest administrable dose according to commercially available formulations.

Pain assessments were performed at the following predefined time points: D0baseline (30 min before premedication), D0pre (immediately before the first oral administration of the assigned treatment), D0post (8 h after the first oral administration), and twice daily thereafter during postoperative days 1 to 5 (morning and afternoon evaluations). An immediate postoperative clinical assessment was performed solely for patient monitoring and clinical decision-making and was not included in statistical comparisons.

Postoperative pain was evaluated using the Dynamic Interactive Visual Analog Scale (DIVAS) (14) and the Glasgow Composite Measure Pain Scale–Short Form (GCMPS-SF) (15). Rescue analgesia (morphine 0.2 mg/kg IV) was administered when GCMPS-SF > 6 or DIVAS > 4.

Dogs remained hospitalized for 5 days with ad libitum access to food and water. During this period, animals were closely monitored for food intake, vomiting, diarrhea, and overall clinical status. All dogs received standardized postoperative antimicrobial therapy consisting of enrofloxacin (10 mg/kg SC) once daily during hospitalization and for 5 days after discharge.

During anesthesia, respiratory rate, end-tidal CO₂ (ETCO₂), inspired CO₂, inspired oxygen fraction (FiO₂), and inhaled and expired isoflurane concentrations were monitored using a capnograph and gas analyzer (R300-RZ, Brazil). Arterial oxygen saturation (SpO₂) was continuously recorded using a pulse oximeter (DX 2022+, Brazil). Arterial blood pressure was measured invasively via catheterization of the pedal artery connected to a multiparameter monitor and continuously monitored along with electrocardiography.

Physiological variables were recorded at 5-min intervals during anesthesia. After extubation, heart rate, respiratory rate, noninvasive blood pressure, and temperature were recorded hourly. Duration of surgery and time to extubation were also recorded.

### 2.4 Cortisol analysis

Venous blood samples were collected for serum cortisol measurement at predefined time points (baseline and postoperative evaluations). Samples were processed by a commercial veterinary diagnostic laboratory (Provet, São Paulo, Brazil).

### 2.5 Statistical analysis

Data distribution was assessed using the Shapiro–Wilk and Lilliefors tests. Homogeneity of variances was evaluated using Bartlett’s test. Data were expressed as mean ± standard deviation (SD). Variables with normal distribution were analyzed using two-way repeated-measures ANOVA to evaluate the effects of treatment group and time, followed by Tukey’s *post hoc* test when appropriate. Assumptions for parametric analysis, including normality and homogeneity of variances, were assessed before repeated-measures ANOVA. When these assumptions were not met, data were transformed when appropriate or analyzed using nonparametric methods. Variables without normal distribution were analyzed using Friedman’s test followed by Dunn’s multiple comparisons test. For repeated-measures outcomes, all animals completed the study and no missing data were present; therefore, no imputation procedures were required. Categorical variables, including the requirement for rescue analgesia, were compared using Fisher’s exact test. When applicable, adjustments for multiple comparisons were performed to control the Type I error rate. Serum cortisol data were transformed (y^0.13) when necessary to satisfy the assumptions of parametric analysis. Original (non-transformed) means are presented in the tables and figures. All statistical analyses were performed using the software R 4.1.0 (R CORE TEAM, 2021). Statistical significance was set at *p* < 0.05 (SD).

**Figure 1:**
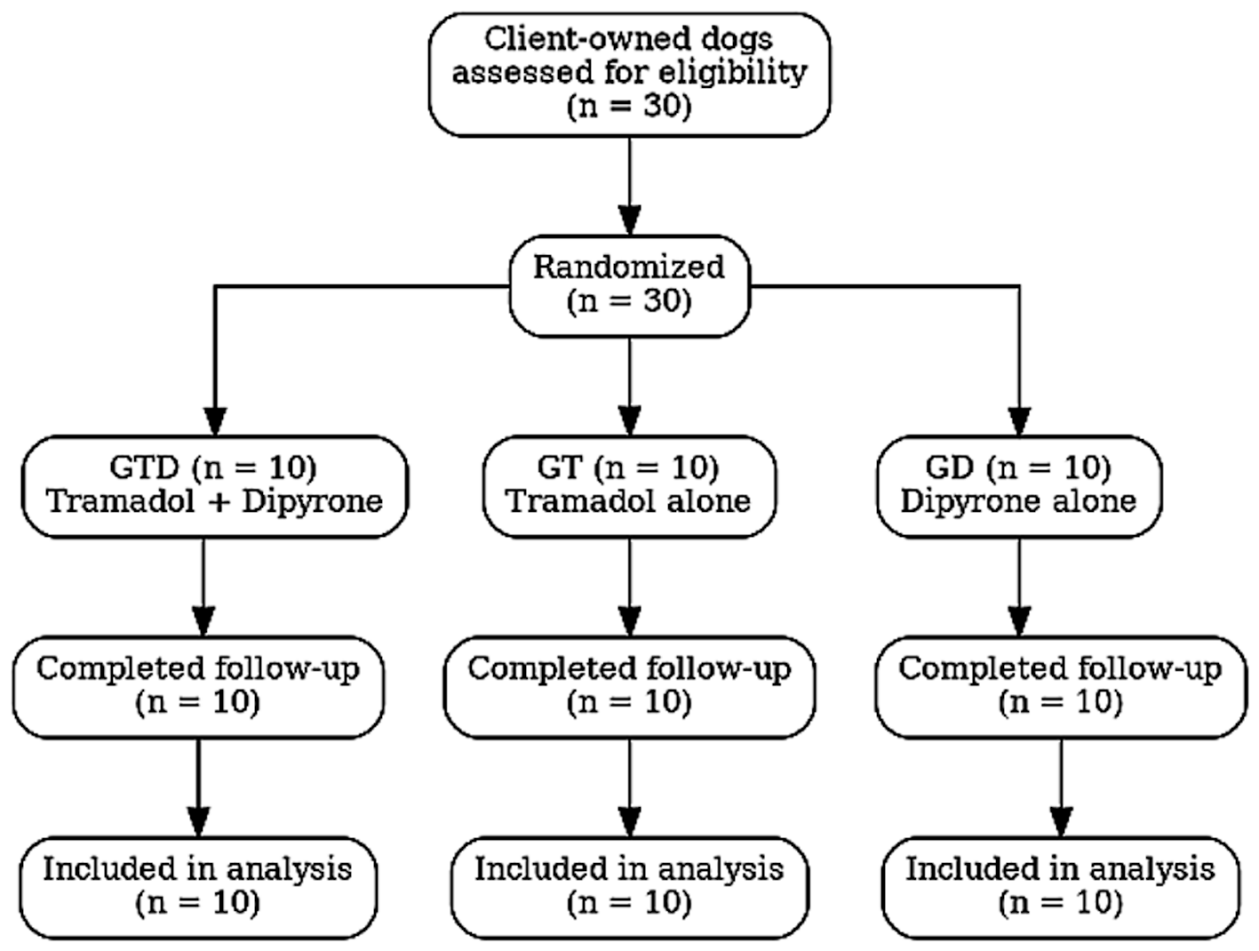
CONSORT flow diagram illustrating the enrollment, randomization, follow-up, and analysis of client-owned dogs included in the study. All randomized dogs completed the study and were included in the final analysis.

The flow of enrolled dogs throughout the study is summarized in the CONSORT flow diagram (Figure 1).

## 3 Results

### 3.1 Demographic and surgical characteristics

Thirty dogs completed the study and were included in the analysis, with 10 animals in each treatment group.

Surgical duration and extubation time did not differ among groups (*p* = 0.29 and *p* = 0.78, respectively). Mean surgical time was 56.4 ± 11.6 min (GTD), 48.8 ± 9.1 min (GT), and 54.5 ± 12.6 min (GD). Extubation times were also comparable, with means of 4.9 ± 2.0, 5.3 ± 1.4, and 5.4 ± 2.3 min, respectively (Table 1).

**Table 1:** Demographic and perioperative characteristics of dogs treated with tramadol–dipyrone combination (GTD), tramadol (GT), or dipyrone (GD).

Variable	Group			
	GTD	GT	GD	*p*-value
Age (years)	8.7 ± 2.6	9.2 ± 1.5	8.9 ± 2.7	0.88
Body weight (kg)	12.4 ± 7.8	8.3 ± 4.4	11.7 ± 9.7	0.46
Surgical duration (min)	56.4 ± 11.6	48.8 ± 9.1	54.5 ± 12.6	0.29
Extubation time (min)	4.9 ± 2.0	5.3 ± 1.4	5.4 ± 2.3	0.78

Values are presented as mean ± SD.

### 3.2 Pain assessment

Postoperative pain scores according to the GCMPS-SF scale peaked immediately after surgery (D0 post) in all groups. At this time point, GTD presented significantly lower pain scores than GD, whereas GT showed intermediate values. On D + 1 morning, GTD maintained significantly lower pain scores than GT, while GD again showed intermediate values (Table 2). Thereafter, pain scores progressively decreased in all groups, and significant differences among treatments were no longer observed consistently throughout the postoperative period.

**Table 2:** Postoperative pain scores (GCMPS-SF) dogs treated with tramadol–dipyrone combination (GTD), tramadol alone (GT), or dipyrone alone (GD) at baseline (D0) and during the postoperative period.

Time points	Mean scores (GCPS-SF)		
	Groups		
	GTD	GT	GD
D0_baseline_	0.5^b^ ± 1.1	0.7^bc^ ± 1.3	1.6^bcd^ ± 1.5
D0_before*_	5.6^a^ ± 2.8	6.5^a^ ± 3.3	4.5^a^ ± 2.5
D0_after**_	1.5^b^ ± 2.2	2.8^bc^ ± 2.9	2.3^abcd^ ± 2.1
D + 1_morning_	0.9^b^ ± 1.7	2.3^bc^ ± 2.5	1.6^bcd^ ± 1.6
D + 1_afternoon_	0.8^Bb^ ± 1.1	1.8^ABbc^ ± 1.7	3.4^Aabc^ ± 3.3
D + 2 _morning_	0.2^b^ ± 0.6	1.4^bc^ ± 2.1	2.0^abcd^ ± 2.4
D + 2 _afternoon_	0.8^b^ ± 1.2	1.0^bc^ ± 1.7	1.3^bcd^ ± 1.7
D + 3 _morning_	0^b^ ± 0.0	1.4^bc^ ± 2.1	0.7^cd^ ± 1.3
D + 3 _afternoon_	0.2^b^ ± 0.4	1.0^bc^ ± 1.5	0.5^d^ ± 1.3
D + 4 _morning_	0.9^b^ ± 1.6	1.0^bc^ ± 1.7	0.8^cd^ ± 1.6
D + 4 _afternoon_	0.2^b^ ± 0.6	1.0^bc^ ± 2.1	0.9^bcd^ ± 1.7
D + _morning_	0.1^b^ ± 0.3	0.2^c^ ± 0.4	0.5^d^ ± 1.3
D + 5 _afternoon_	0.1^b^ ± 0.3	0.3^c^ ± 0.9	0.4^d^ ± 1.3

Values are presented as mean ± SD. Different uppercase letters within the same row indicate significant differences among treatment groups. Different lowercase superscript letters within the same column indicate significant differences over time within the same treatment group (Tukey test, *p* < 0.05). *After surgery and before initiation of oral treatment. **After initiation of oral treatment.

DIVAS scores showed a similar pattern to those observed with the GCMPS-SF. Significant differences among groups were detected during the early postoperative period, particularly at D + 1 afternoon, when dogs receiving dipyrone alone presented higher scores than those treated with the tramadol–dipyrone combination.

### 3.3 Pain incidence and rescue analgesia

Pain incidence analysis according to the GCMPS-SF demonstrated that most dogs were classified as pain-free or having only mild pain during postoperative follow-up (Table 3). Moderate pain was observed predominantly during the immediate postoperative period, particularly in GT, whereas no severe pain was recorded during postoperative monitoring. At D + 1, a significantly higher proportion of dogs without pain was observed in the combination group compared with the dipyrone group (*p* < 0.05). From D + 2 onward, most animals in all groups were classified as having no detectable pain.

**Table 3:** Postoperative pain incidence (100%) according to the GCMPS-SF in dogs treated with tramadol–dipyrone combination (GTD), tramadol alone (GT), or dipyrone alone (GD).

Time points	Pain incidence														
	GTD					GT					GD				
	No pain	Mild	Moderate	Intense/Severe		No pain	Mild	Moderate	Intense/Severe		No pain	Mild	Moderate	Intense/Severe	
D0_baseline_	10 (100)	0	0	0	b	10 (100)	0	0	0	b	10 (100)	0	0	0	b
D0_before*_	2 (20)	4 (40)	4 (40)	0	a	2 (20)	3 (30)	5 (50)	0	a	2 (20)	6 (60)	2 (20)	0	a
D0_after**_	7 (70)	3 (30)	0	0	ab	6 (60)	2 (20)	2 (20)	0	ab	7 (70)	3 (30)	0	0	ab
D + 1_morning_	9 (90)	1 (10)	0	0	b	6 (60)	4 (40)	0	0	ab	8 (80)	2 (20)	0	0	ab
D + 1_afternoon_	10 (100)	0	0	0	Ab	9 (90)	1 (10)	0	0	ABab	5 (50)	3 (30)	2 (20)	0	Ba
D + 2 _morning_	10 (100)	0	0	0	b	8 (80)	2 (20)	0	0	ab	6 (60)	4 (40)	0	0	ab
D + 2 _afternoon_	10 (100)	0	0	0	b	9 (90)	1 (10)	0	0	ab	8 (80)	2 (20)	0	0	ab
D + 3 _morning_	10 (100)	0	0	0	b	8 (80)	2 (20)	0	0	ab	9 (90)	1 (10)	0	0	ab
D + 3_afternoon_	10 (100)	0	0	0	b	9 (90)	1 (10)	0	0	ab	9 (90)	1 (10)	0	0	ab
D + 4 _morning_	9 (90)	1 (10)	0	0	b	9 (90)	1 (10)	0	0	ab	9 (90)	1 (10)	0	0	ab
D + 4_afternoon_	10 (100)	0	0	0	b	8 (80)	2 (20)	0	0	ab	9 (90)	1 (10)	0	0	ab
D + _morning_	10 (100)	0	0	0	b	10 (100)	0	0	0	b	9 (90)	1 (10)	0	0	ab
D + 5_afternoon_	10 (100)	0	0	0	b	10 (100)	0	0	0	b	9 (90)	1 (10%)	0	0	ab

Data are presented as *n* (%). Different uppercase letters within the same row indicate significant differences among treatment groups. Different lowercase superscript letters within the same column indicate significant differences over time within the same treatment group (Tukey test, *p* < 0.05). *After surgery and before initiation of oral treatment. **After initiation of oral treatment.

Before initiation of oral treatment, rescue analgesia was required in 40, 50, and 20% of dogs in the tramadol–dipyrone, tramadol, and dipyrone groups, respectively. After initiation of oral treatment, rescue analgesia was no longer required in the GTD, whereas it was required in 20% of dogs in both the tramadol and dipyrone groups.

No significant differences among groups were observed within each time point (*p* > 0.05). However, a significant time effect was observed in the combination and tramadol groups (*p* < 0.05), indicating a reduction in rescue analgesia after initiation of treatment. The cumulative morphine dose administered as rescue analgesia did not differ among treatment groups.

### 3.4 Serum cortisol concentrations

Serum cortisol concentrations differed among groups on D + 1 morning (*p* < 0.01), with GT showing significantly higher values than GTD, while GD showed intermediate values (Table 4). Mean cortisol concentrations in GT and GD transiently exceeded the laboratory reference range (1.0–4.6 μg/dL), whereas values in GTD remained within physiological limits throughout the study period. No significant effect of time was detected within groups (*p* = 0.2824).

**Table 4:** Serum cortisol concentrations (μg/dL) in dogs treated with the tramadol–dipyrone combination (GTD), tramadol alone (GT), or dipyrone alone (GD) at baseline (D0) and on postoperative days 1–5.

	Group		
Time point	GTD	GT	GD
Baseline (D0)	1.6 ± 0.7	2.7 ± 1.8	2.9 ± 2.5
D + 1 morning	3.3 ± 2.3ᵇ	6.4 ± 4.3ᵃ	4.7 ± 1.9ᵃᵇ
D + 2 morning	2.1 ± 0.9	3.4 ± 1.7	2.8 ± 1.0
D + 3 morning	2.5 ± 1.5	3.5 ± 2.6	2.0 ± 1.0
D + 4 morning	2.9 ± 1.2	2.4 ± 0.9	2.6 ± 1.2
D + 5 morning	1.9 ± 1.5	3.8 ± 3.2	2.6 ± 1.6

Values are presented as mean ± SD. Different superscript letters within the same row indicate significant differences among groups (Tukey test, *p* < 0.05). Cortisol was measured before surgery (Baseline, D0) and on the morning of postoperative days 1–5. Reference interval for serum cortisol: 1.0–4.6 μg/dL (Provet, São Paulo, Brazil).

### 3.5 Physiological parameters and adverse effects

Physiological parameters including heart rate, systolic arterial pressure, respiratory rate, and body temperature showed expected postoperative variations and remained within physiological reference ranges throughout the study in all groups. Although isolated statistically significant differences among treatments were observed at some time points, these differences showed no consistent temporal pattern and were not considered clinically relevant. Body temperature decreased after surgery in all groups, consistent with the expected effects of anesthesia and surgical exposure.

No clinically relevant gastrointestinal signs or changes in food or water intake were observed, and all dogs maintained normal postoperative recovery throughout the monitoring period.

## 4 Discussion

Effective postoperative analgesia is a fundamental component of perioperative care in veterinary patients, particularly in procedures associated with moderate to severe tissue trauma. In the present study, dogs undergoing ovariohysterectomy combined with unilateral mastectomy received a standardized multimodal perioperative analgesic protocol consisting of preoperative opioid administration (meperidine), postoperative anti-inflammatory therapy (meloxicam), and group-specific analgesic treatments (dipyrone, tramadol, or the tramadol–dipyrone combination). Although all protocols contributed to postoperative analgesia, rescue analgesia was required in a proportion of dogs during the early postoperative period, highlighting the substantial analgesic demands associated with ovariohysterectomy combined with unilateral mastectomy. Nevertheless, the tramadol–dipyrone combination resulted in improved early postoperative pain control. The consistency between DIVAS and GCMPS-SF findings strengthens the interpretation that the tramadol–dipyrone combination provided superior analgesic control during the early postoperative period. This multimodal approach reflects current recommendations for perioperative pain management and was designed to provide broad analgesic coverage during the immediate postoperative period (1, 2).

It should also be considered that all dogs received a standardized anesthetic and analgesic protocol that included preoperative meperidine and intraoperative fentanyl administration (16). Although residual effects of these agents may have contributed to analgesia during the immediate postoperative period, their administration was identical across groups and therefore is unlikely to explain the differences observed after initiation of oral treatment.

A recent systematic review evaluating perioperative analgesic strategies in dogs undergoing castration procedures demonstrated that multimodal protocols combining opioids and NSAIDs are associated with a markedly lower need for rescue analgesia compared with isolated analgesic treatments (4). In the present study, dipyrone was incorporated into the multimodal protocol considering the greater surgical trauma associated with unilateral mastectomy combined with ovariohysterectomy compared with routine gonadectomy procedures (17).

Although pain scores decreased substantially in all groups after initiation of oral treatment, the transient differences observed during the early postoperative phase are clinically relevant. The decrease in pain scores between D0pre and D0post likely reflects not only the expected postoperative reduction in nociceptive input over time but also the contribution of the instituted multimodal analgesic protocol. The first 24 h after surgery represent the period during which nociceptive input and inflammatory responses are most intense, and inadequate analgesia during this interval may contribute to increased physiological stress and delayed recovery (18). Dogs receiving the tramadol–dipyrone combination showed improved pain control during this early postoperative period. Serum cortisol concentrations provided additional support for the clinical findings observed during the early postoperative period. Although cortisol is a nonspecific biomarker influenced by multiple physiological and environmental factors, increases in circulating concentrations are commonly associated with surgical stress, pain, and activation of the hypothalamic–pituitary–adrenal axis (17, 19, 20). In the present study, dogs receiving the tramadol–dipyrone combination maintained cortisol concentrations within the laboratory reference range, whereas transient increases above reference values were observed in the tramadol and dipyrone groups. These findings are consistent with the pain assessment results and may reflect improved modulation of the postoperative stress response in dogs receiving combination therapy.

Although differences in rescue analgesia among groups were not statistically significant, no dog in the GTD group required rescue analgesia after initiation of oral treatment whereas rescue analgesia was still required in both the tramadol and dipyrone groups. Although this finding should be interpreted cautiously given the sample size, it suggests that the combination therapy may have provided more consistent postoperative pain control at the individual patient level. However, it is important to emphasize that all dogs received meloxicam as part of a multimodal analgesic protocol. Therefore, the present findings should be interpreted as a comparison between clinically applicable multimodal postoperative analgesic strategies rather than an evaluation of tramadol or dipyrone administered as sole analgesic agents. All dogs also received the same standardized anesthetic protocol, including intraoperative fentanyl administration. Although residual opioid effects may have contributed to analgesia during the immediate postoperative period, this potential influence was identical across treatment groups and is therefore unlikely to have influenced the between-group diferences observed after initiation of the oral treatments. Although the magnitude of the observed analgesic benefit was relatively small and dipyrone alone provided satisfactory analgesia in most dogs, the combination therapy was associated with improved pain control during the early postoperative period, when nociceptive input and inflammatory responses are expected to be greatest. Because a minimal clinically important difference has not been established for the Glasgow Composite Measure Pain Scale–Short Form in dogs undergoing this type of surgical procedure, the clinical relevance of the findings was interpreted by integrating multiple complementary outcome measures, including behavioral pain scores, rescue analgesia requirements, and serum cortisol concentrations. The relatively small sample size may have limited the statistical power to detect differences among treatments.

The relatively high frequency of rescue analgesia observed during the immediate postoperative period reinforces the importance of close postoperative monitoring in dogs undergoing procedures associated with substantial tissue trauma. Although all animals received multimodal perioperative analgesia, additional opioid administration was required in a proportion of dogs during the first hours after surgery, highlighting the intense nociceptive and inflammatory stimulation associated with ovariohysterectomy combined with unilateral mastectomy. These findings suggest that supplemental opioid analgesia may be necessary in some patients during the immediate postoperative period, even when multimodal analgesic protocols are employed.

The interpretation of tramadol efficacy in dogs remains controversial, largely because this species exhibits variable and often limited formation of the active metabolite O-desmethyltramadol (M1), which contributes substantially to *μ*-opioid receptor-mediated analgesia (5, 21). However, the clinical effects of tramadol cannot be interpreted exclusively based on M1 production, as tramadol also exerts antinociceptive effects through inhibition of serotonin and noradrenaline reuptake pathways (22). Consequently, when incorporated into a multimodal analgesic protocol, tramadol may contribute to postoperative analgesia through complementary mechanisms even if its opioid-mediated effects are less pronounced than those observed in other species.

Accordingly, the contribution of tramadol should be interpreted within the broader context of multimodal analgesia and potential pharmacodynamic interactions rather than as a stand-alone analgesic.

Previous clinical studies support the use of tramadol as part of multimodal analgesic strategies in dogs. Monteiro et al. (9) demonstrated improved analgesic outcomes when tramadol was combined with ketoprofen in dogs with osteoarthritis. Similarly, Martins et al. reported effective pain control when tramadol or codein were administered together with ketoprofen, with additional improvement following rescue dipyrone administration (23). Clinical data from oncologic pain patients have also suggested beneficial effects when tramadol is incorporated into multimodal analgesic protocols (7). Furthermore, a recent systematic review and meta-analysis concluded that tramadol may reduce the need for rescue analgesia compared with placebo or no treatment, although the overall quality of evidence was considered low to very low because of methodological heterogeneity and imprecision across studies (24). In contrast, Domínguez-Oliva et al. highlighted that, despite variability in opioid-mediated effects, tramadol may still provide clinically relevant analgesia in dogs when appropriately dosed and incorporated into multimodal analgesic protocols (25).

Recent clinical investigations also support the analgesic potential of tramadol in dogs undergoing surgical procedures. Tettrene et al. reported lower postoperative pain scores and fewer rescue interventions in dogs treated with tramadol compared with morphine following ovariohysterectomy (26). Similarly, Di Salvo et al. (27) found that tramadol administered either intravenously or intranasally provided postoperative analgesia comparable to methadone, while Cicirelli et al. reported analgesic outcomes similar to those observed with transdermal fentanyl during the first 24 h after ovariectomy and mastectomy (28). However, not all studies have reached similar conclusions. Several experimental and clinical investigations have failed to demonstrate consistent clinically relevant analgesic effects of tramadol in dogs, findings that have largely been attributed to limited M1 formation, methodological differences among studies, and variability in pain models (29).

It is also important to consider that tramadol exhibits dose-dependent analgesic efficacy in dogs. While doses of approximately 4-5 mg/kg have frequently been used in studies evaluating tramadol as a sole analgesic (8, 28), the present study employed a lower tramadol dose (2 mg/kg) combined with dipyrone (25 mg/kg). The rationale for this approach was to explore potential synergistic interactions between these drugs while minimizing opioid-related adverse effects.

In a recent pharmacokinetic study conducted by Mouta et al. (30), the association between dipyrone and tramadol in dogs demonstrated relevant pharmacokinetic interactions characterized by increased AUC, reduced clearance, and prolonged persistence of the dipyrone metabolite 4-aminoantipyrine (AA). In contrast, reductions in 4-methylaminoantipyrine (MAA) Cmax and C0 occurred without changes in overall AUC, suggesting lower peak plasma concentrations while maintaining global exposure. This profile may contribute to more stable drug exposure and potentially reduce concentration-dependent adverse effects.

Comparison with previous studies evaluating tramadol alone demonstrated that M1 concentrations remained consistent with values previously reported in dogs, reflecting the known limited formation of this active metabolite in the species (5, 31). However, tramadol also showed a tendency toward prolonged systemic persistence, supported by increases in parameters such as half-life and mean residence time, suggesting possible modulation of tramadol pharmacokinetics when administered in combination with dipyrone.

Collectively, these findings support the hypothesis that the tramadol–dipyrone association may prolong exposure to both tramadol and the active dipyrone metabolite AA, potentially contributing to prolonged analgesic effects and modulation of the postoperative analgesic profile observed in the present study. Dipyrone represents an interesting partner in such combinations because its central and peripheral antinociceptive mechanisms differ from those of both opioids and NSAIDs (32). When used as a sole analgesic in dogs and cats undergoing ovariohysterectomy, dipyrone is frequently associated with rescue analgesia rates ranging from approximately 20 to 40%, depending on the dose and protocol employed (33, 34). In a recent clinical trial conducted by our group in cats, the association of tramadol and dipyrone substantially reduced the need for rescue analgesia, with most animals not requiring additional postoperative analgesic intervention (13). Together, these findings support the concept that dipyrone may contribute more effectively when incorporated into a multimodal analgesic strategy than when used as a single postoperative analgesic.

Finally, from a clinical perspective, the availability of effective oral analgesic options remains particularly important for postoperative management in veterinary patients. In many clinical settings, dogs are discharged shortly after surgery, and analgesic treatment must be continued at home by caregivers. Moreover, in countries such as Brazil, tramadol and dipyrone are among the most accessible and widely prescribed oral analgesics for dogs (35). The availability of a fixed-dose combination may therefore facilitate postoperative pain management by simplifying administration, improving treatment adherence, and maintaining multimodal analgesic coverage during the recovery period. Additionally, both drugs are generally associated with a favorable tolerability profile, making this combination particularly attractive for home use (36, 37).

The relatively extended postoperative monitoring period used in this study also allowed the detection of potential delayed adverse effects and provided a more comprehensive evaluation of postoperative analgesic efficacy than studies limited to the immediate postoperative period.

Overall, the evaluated protocols were well tolerated. No clinically relevant hemodynamic instability or body temperature reduction was observed, supporting the safety of dipyrone in the postoperative setting, including when used in combination therapy. Importantly, no clinically relevant gastrointestinal adverse effects or reductions in food or water intake were observed, further supporting its tolerability. These findings are particularly relevant considering the potential effects of prostaglandin inhibition on gastrointestinal function, which were not clinically evident in the present study.

Some limitations of the present study should be acknowledged. The number of animals included in each treatment group was relatively small and no *a priori* sample size calculation was performed. Although clinically relevant differences were observed among treatments, particularly regarding early postoperative pain scores, the limited sample size may have reduced the statistical power to detect differences in some secondary outcomes such as rescue analgesia. In addition, despite the use of validated behavioral pain scales and physiological indicators, the assessment of pain in animals remains inherently challenging. Nevertheless, the use of multiple complementary outcome measures provided a comprehensive evaluation of analgesic efficacy.

Another limitation of the present study was the absence of a placebo-treated control group. However, because client-owned dogs underwent unilateral mastectomy combined with ovariohysterectomy, withholding postoperative analgesia was considered ethically unjustifiable and inconsistent with current standards of veterinary clinical care. Furthermore, previous studies have demonstrated that NSAID administration alone does not consistently provide adequate postoperative analgesia following ovariohysterectomy and other moderate-to-severe surgical procedures in dogs, supporting the routine use of multimodal analgesic protocols (4, 38). Therefore, the present study was designed to compare clinically applicable postoperative analgesic strategies rather than analgesia versus no treatment. Finally, as the study was conducted in a single hospital with one surgeon and within one geographic region, the generalizability of the findings may be limited. Future multicenter studies will be necessary to validate these results on a broader scale.

## 5 Conclusion

The results of the present study suggest that the tramadol–dipyrone combination may represent a useful component of multimodal postoperative analgesia in dogs undergoing surgical procedures associated with significant tissue trauma. Although the observed differences were relatively small, the combination was associated with improved early postoperative pain control and, notably, no dogs required rescue analgesia after initiation of oral treatment, while maintaining good tolerability throughout the monitoring period.

The availability of this combination as an oral formulation may represent a practical option for maintaining multimodal analgesia during the postoperative recovery period, particularly after hospital discharge when continued pain management becomes the responsibility of caregivers. Further studies including larger populations are warranted to better define the role of this association within perioperative analgesic protocols in dogs.

## Data Availability

The raw data supporting the conclusions of this article will be made available by the authors, without undue reservation.
